# Measurements in Dementia Care and Outcomes: Advancing Research and Practice With Innovative Methods

**DOI:** 10.1093/geroni/igaf122.1334

**Published:** 2025-12-31

**Authors:** Anju Paudel, Wen Liu, Ann Kolanowski

## Abstract

Many individuals living with dementia in the U.S. receive care and support across settings of care. Growing research has focused on optimizing the quality of dementia care and outcomes through behavioral interventions and utilizing validated measures to assess the impact of behavioral interventions on quality of care and outcomes. This symposium describes different methodologies (Delphi Survey, Tool Modification, Structural validation, Electronic Medical Record Validation) to develop and validate measures assessing important aspects of dementia care and outcomes (care interactions, mealtime behaviors, rejection of care, and pain) in nursing home (NH), hospital, and assisted living (AL) settings. The first paper describes the development and refinement of the Quality of Interactions Inventory (QUALII) tool that assesses positive care interactions in dementia care in ALs. The second paper discusses the development, validity, and reliability of the Pain Competency Evaluation in Dementia (PACED) scale in NH. Next, the adaptation of an existing tool - Cue Utilization and Engagement in Dementia (CUED) - into three observational scales to assess NH residents’ mealtime behaviors is presented. The fourth paper describes the Modified Quality of Interactions Schedule (MQuIS) that assesses staff-resident interactions and differences in staff-resident interactions by racial concordance versus discordance in ALs. The final presentation describes validation of a practical protocol using electronic medical record nurse charting data against a gold-standard tool of rejection of care. We will compare the methodologies based on research questions, identify directions for future research in measurements in dementia care and outcomes, and explore practice implications of discussed measures.

